# Peptidoglycan-type analysis of the *N*-acetylmuramic acid auxotrophic oral pathogen *Tannerella forsythia* and reclassification of the peptidoglycan-type of *Porphyromonas gingivalis*

**DOI:** 10.1186/s12866-019-1575-7

**Published:** 2019-09-02

**Authors:** Valentina M. T. Mayer, Isabel Hottmann, Rudolf Figl, Friedrich Altmann, Christoph Mayer, Christina Schäffer

**Affiliations:** 1 Department of NanoBiotechnology, NanoGlycobiology unit, Universität für Bodenkultur Wien, Vienna, Austria; 2 Department of Biology, Interfaculty Institute of Microbiology and Infection Medicine, Eberhard Karls Universität, Tübingen, Germany; 3 Department of Chemistry, Institute of Biochemistry, Universität für Bodenkultur Wien, Vienna, Austria

**Keywords:** *Tannerella forsythia*, Peptidoglycan, Radioassay, Diaminopimelic acid, *Porphyromonas gingivalis*

## Abstract

**Background:**

*Tannerella forsythia* is a Gram-negative oral pathogen. Together with *Porphyromonas gingivalis* and *Treponema denticola* it constitutes the “red complex” of bacteria, which is crucially associated with periodontitis, an inflammatory disease of the tooth supporting tissues that poses a health burden worldwide. Due to the absence of common peptidoglycan biosynthesis genes, the unique bacterial cell wall sugar *N*-acetylmuramic acid (MurNAc) is an essential growth factor of *T. forsythia* to build up its peptidoglycan cell wall. Peptidoglycan is typically composed of a glycan backbone of alternating *N*-acetylglucosamine (GlcNAc) and MurNAc residues that terminates with anhydroMurNAc (anhMurNAc), and short peptides via which the sugar backbones are cross-linked to build up a bag-shaped network.

**Results:**

We investigated *T. forsythia’s* peptidoglycan structure, which is an essential step towards anti-infective strategies against this pathogen. A new sensitive radioassay was developed which verified the presence of MurNAc and anhMurNAc in the cell wall of the bacterium. Upon digest of isolated peptidoglycan with endo-*N*-acetylmuramidase, exo-*N*-acetylglucosaminidase and muramyl-L-alanine amidase, respectively, peptidoglycan fragments were obtained. HPLC and mass spectrometry (MS) analyses revealed the presence of GlcNAc-MurNAc-peptides and the cross-linked dimer with retention-times and masses, respectively, equalling those of control digests of *Escherichia coli* and *P. gingivalis* peptidoglycan. Data were confirmed by tandem mass spectrometry (MS^2^) analysis, revealing the GlcNAc-MurNAc-tetra-tetra-MurNAc-GlcNAc dimer to contain the sequence of the amino acids alanine, glutamic acid, diaminopimelic acid (DAP) and alanine, as well as a direct cross-link between DAP on the third and alanine on the fourth position of the two opposite stem peptides. The stereochemistry of DAP was determined by reversed-phase HPLC after dabsylation of hydrolysed peptidoglycan to be of the *meso*-type.

**Conclusion:**

*T. forsythia* peptidoglycan is of the A1γ-type like that of *E. coli*. Additionally, the classification of *P. gingivalis* peptidoglycan as A3γ needs to be revised to A1γ, due to the presence of *meso*-DAP instead of LL-DAP, as reported previously.

**Electronic supplementary material:**

The online version of this article (10.1186/s12866-019-1575-7) contains supplementary material, which is available to authorized users.

## Background

The anaerobic, Gram-negative oral pathogen *Tannerella forsythia* is affiliated to the *Bacteroidetes* phylum of bacteria and plays an essential role in the onset and progression of periodontitis [1]. Periodontitis is a chronic, inflammatory disease of the tooth-supporting tissues, which crucially involves the so-called “red complex” consortium of bacteria, comprising the phylogenetically related bacteria *T. forsythia* (previously, *Bacteroides forsythus* [2]) and *Porphyromonas gingivalis* (previously, *Bacteroides gingivalis* [3]) and the spirochete *Treponema denticola*, which act as late colonizers within subgingival plaque biofilms [4, 5]. *T. forsythia* has a strict auxotrophy for the unique bacterial cell wall aminosugar *N*-acetylmuramic acid (MurNAc) and changes its morphology under MurNAc depletion from rod-shaped to enlarged, fusiform cells under laboratory cultivation conditions [6, 7]. Due to the absence of the common peptidoglycan (PGN) biosynthesis genes *murA* and *murB* [8], encoding a UDP-*N*-acetylglucosamine 1-carboxyvinyltransferase and a UDP-*N*-acetylenolpyruvoyl-glucosamine reductase, respectively, yielding UDP-MurNAc from UDP-*N*-acetylglucosamine glucose-1-phosphate, MurNAc needs to be externally provided and a novel bypass route for the synthesis of UDP-MurNAc [9] is proposed for *T. forsythia*. In its natural habitat, the oral cavity, *T. forsythia* covers its MurNAc requirements mainly by scavenging of cohabiting bacteria [10].

Considering that *T. forsythia* cannot de novo synthesise PGN and several recent studies are dealing with the PGN metabolism of this bacterium - addressing aspects such as the identification of a novel MurNAc transporter [11] as well as the regulation and environmental muropeptide uptake and utilization [12] - it is surprising that the composition and structure of *T. forsythia’s* PGN are still unknown. PGN is a macromolecule, forming a huge net-shaped structure (known as PGN sacculus) that encases the entire bacterial cell and is required for protection against adverse environmental effects and to maintain the cell shape [13]. While Gram-positive bacteria possess a thick PGN layer external to the cytoplasmic membrane, Gram-negatives have a thin, possibly monolayered structure, which is covalently linked to the outer membrane through the prominent Braun’s lipoprotein [14].

On the primary structure level, PGN comprises a backbone of alternating β-1,4-linked *N*-acetylglucosamine (GlcNAc) and MurNAc residues, terminating with a non-reducing 1,6-anhydroMurNAc (anhMurNAc) residue [15–17]. These linear glycan chains are cross-linked via short peptides, which are bound to the free carboxylic acid of the lactyl substituent of MurNAc. The lengths of glycan and peptide chains and the amount of cross-linkage can differ within growth phases. For the Gram-negative bacterium *Escherichia coli*, it was reported that the GlcNAc-MurNAc-tetrapeptide (G-M-tetra) represents about 30% of the total material, followed by the cross-linked disaccharide tetra-tetrapeptide (G-M-tetra-tetra-M-G), with the presence of tripeptide fractions increasing in the stationary growth phase [14, 18–20]. Although PGN sacculi are able to withstand high osmotic pressure, the structure is extremely flexible and enables the diffusion of proteins [18]. Based on conformational energy calculations, the glycan backbone is assumed to be rather rigid, whereas the stem peptides are assumed to be the flexible part of the structure [21]. To date, it has been impossible to obtain a crystal structure of PGN and, thus, its three-dimensional architecture remains unknown. However, several hypothetical PGN models are available. The so-called “scaffold” model proposes glycan strands protruding vertically from the cytoplasmic membrane [22, 23], however, glycan strands of an average length of 20 disaccharide units and more are too long to coincide with that type of architecture. In recent models of layered PGN, glycans are arranged parallel to the cytoplasmic membrane, forming a monolayer by cross-linkage of peptides of neighbouring strands. This model is in accordance with experimental data showing that approximately 40 to 50% of peptides are part of cross-links in Gram-negatives, or up to 90% in the Gram-positive bacterium *Staphylococcus aureus* [14, 19].

A basic classification of PGN based on the mode of cross-linkage was established by Schleifer and Kandler [15]. Following this scheme, PGN is classified as group A or group B, denoting cross-linkage between the third and the fourth or between the second and the fourth amino acid of two peptide subunits. Further, numbers define subgroups, which are determined by the type of cross-linkage, and Greek letters determine the involved diamino acid. In Gram-negative bacteria, the stem peptide frequently contains L-Ala-*iso*-D-Glu-*m*-DAP-D-Ala-D-Ala (where *m*-DAP is *meso*-diaminopimelic acid), connecting the glycan strands through direct *m*-DAP-D-Ala cross-linkage [14]. This composition, as found in *E. coli*, refers to the PGN-type A1γ [15]. In comparison to the great variety of PGN in Gram-positive organisms, Gram-negative bacteria reveal few variations. Substitution of *m*-DAP by other amino acids like L-ornithine, as reported for *Treponema phagedenis* [24] or lanthionine, as reported for *Fusobacterium nucleatum*, [25] is possible. Additionally, *m*-DAP may be replaced by the stereochemical variant LL-DAP, as was reported for *P. gingivalis* [26].

In this study, the PGN structure of the MurNAc-auxotroph *T. forsythia* was investigated for the first time, by using a combined approach of HPLC and electrospray ionization (ESI)-MS, MS^2^, and a novel radioassay, and its chemical type according to the PGN classification scheme introduced by Schleifer and Kandler was determined [15]. Furthermore, we show that the PGN-type of the phylogenetically related bacterium *P. gingivalis* needs to be revised, due to the presence of *m*-DAP instead of LL-DAP, as reported previously [26].

## Results

### Identification of MurNAc and anhMurNAc in *T. forsythia* cell walls

**Fig. 1:**
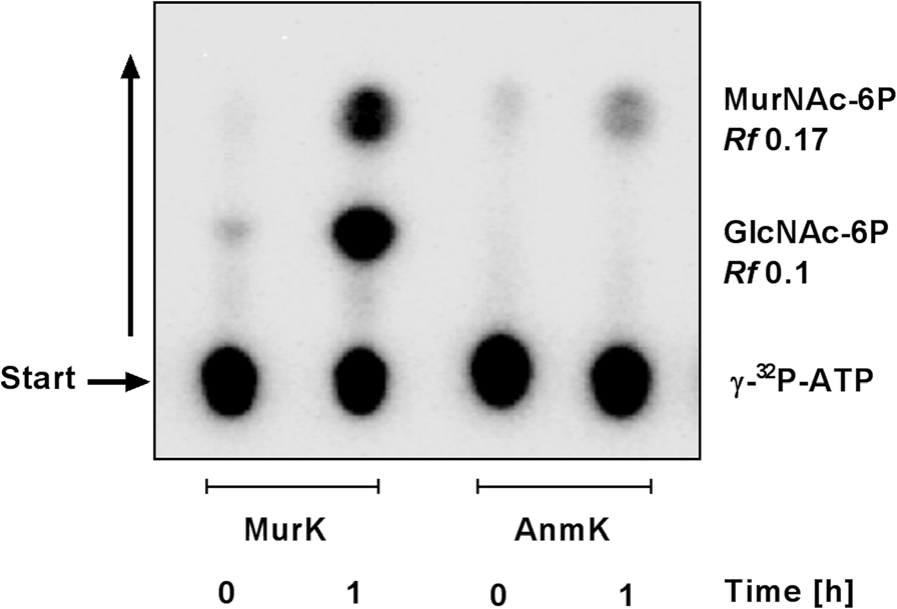
Autoradiogram of a TLC showing radioactive phosphorylation of digested *T. forsythia* cell walls with γ-^*32*^P-ATP by GlcNAc/MurNAc kinase MurK, yielding GlcNAc-6-phosphate (GlcNAc-6^32^P) and MurNAc 6-phosphate (MurNAc-6^32^P), and by anhMurNAc kinase AnmK of *E. coli*, yielding MurNAc-6^32^P. Reaction time points were 0 and 1 h. The representative section of the autoradiogram is shown. The running direction of the solvent is indicated by an upward arrow on the left side of the plate

The development of a new sensitive radioassay for PGN is based on radioactive phosphorylation with γ-^32^P-ATP and activity of recently characterized PGN recycling enzymes. *T. forsythia* cell walls were digested with the endo-*N*-acetylmuramidase mutanolysin, the exo-*N*-acetyl-glucosaminidase NagZ and the muramyl-L-alanine amidase AmiD to produce single PGN sugars. The GlcNAc/MurNAc kinase MurK of *Clostridium acetobutylicum* [27] successfully yielded radioactively labelled GlcNAc-6-phosphate (GlcNAc-6^32^P; *Rf*~ 0.10) and MurNAc 6-phosphate (MurNAc-6^32^P; *Rf*~ 0.17) out of digested *T. forsythia* cell walls, proving the presence of the typical PGN backbone sugars. The presence of anhMurNAc was inferred from radioactive phosphorylation with the anhMurNAc kinase AnmK of *E. coli* [28]*,* yielding MurNAc-6^32^P (Fig. 1). The low amounts of anhMurNAc as detected by autoradiography of the TLC plate might be indicative of extended glycan backbones terminating with anhMurNAc, as typical of Gram-negative bacteria [16, 17].

### Elucidation of typical PGN building blocks by LC-MS

**Fig. 2:**
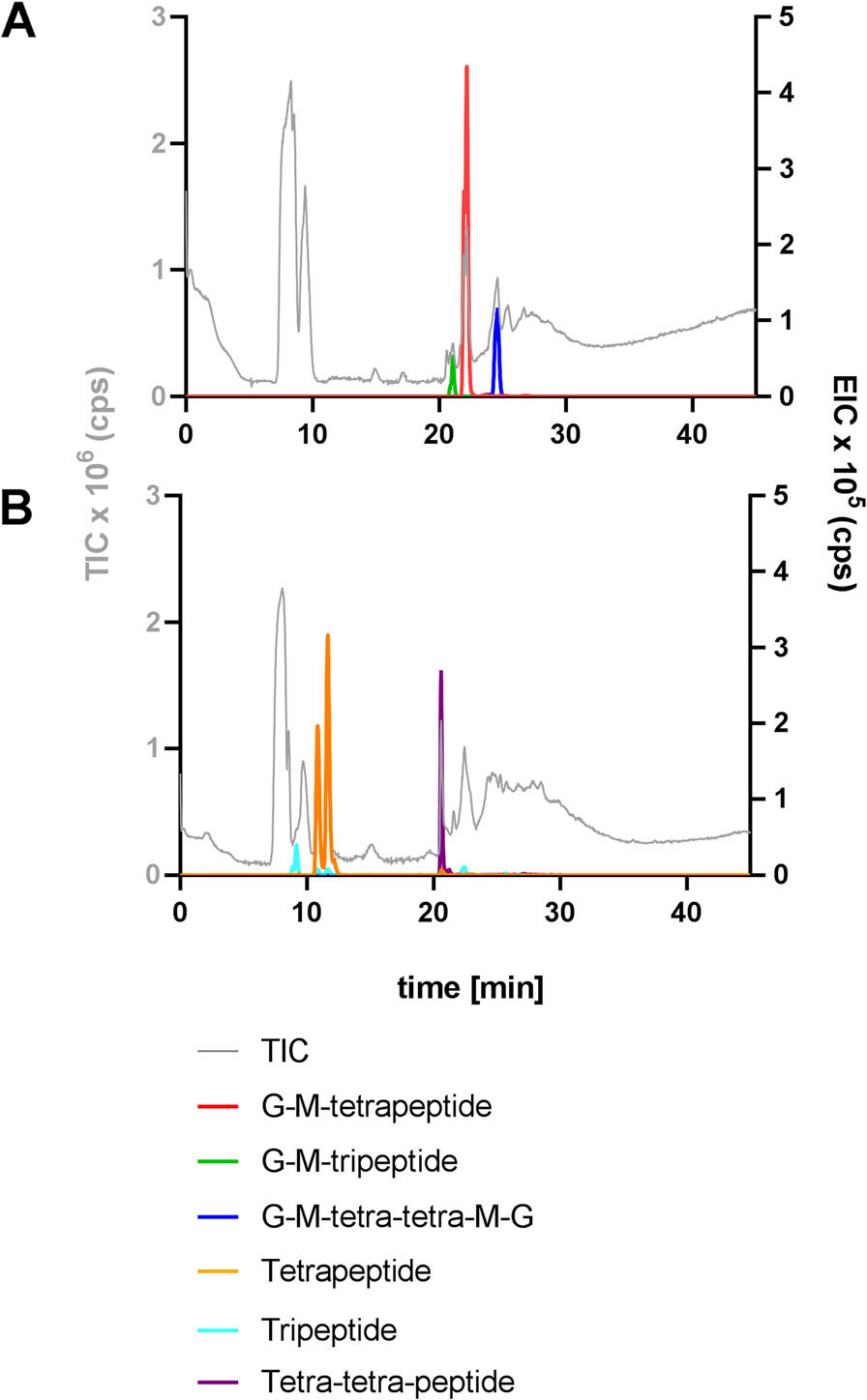
LC-MS analysis of PGN digests with (**a**) mutanolysin yielding GlcNAc-MurNAc-peptides and cross-linked GlcNAc-MurNAc-tetra-tetra-MurNAc-GlcNAc and (**b**) NagZ and amidase, yielding single peptides. Legend: TIC, total ion chromatogram; G, GlcNAc; M, MurNAc

PGN was isolated following a published procedure [29] and digested with the endo-*N*-acetylmuramidase mutanolysin from *S. globisporus*, which cleaves the β-1,4-linkage of the PGN backbone between MurNAc and GlcNAc. Digestion products were reduced, applied to RP-HPLC, and used as peak source for structural analysis. MS measurements revealed the expected presence of GlcNAc-MurNAc-peptides (G-M-tri/tetra), with special regards to the monomers G-M-tri and G-M-tetra and the cross-linked dimer G-M-tetra-tetra-M-G. Theoretical masses of these PGN building blocks with an assumed stem peptide composition of Ala-Glu-DAP(−Ala) were 871.378 *m/z* for G-M-tri, 942.416 *m/z* for G-M-tetra, and 1865.813 *m/z* for G-M-tetra-tetra-M-G. Measured in positive ion mode, the observed peaks were 871.376 *m/z* [M + H]^+^ for G-M-tri, 942.415 *m/z* [M + H]^+^ for G-M-tetra, and 933.414 *m/z* [M + 2H]^2+^ for double charged G-M-tetra-tetra-M-G and, thus, in accordance with theoretical masses (Fig. 2).

This was confirmed by a further digest with the *N*-acetylglucosaminidase NagZ and the muramyl-L-alanine amidase AmiD, which successfully produced single peptides out of GlcNAc-MurNAc-peptides. Observed *m/z* values in positive ion mode were 391.181 *m/z* [M + H]^+^ for tripeptide, 462.221 *m/z* [M + H]^+^ for tetrapeptide, and 905.417 *m/z* [M + H]^+^ for the tetra-tetra-peptide, conforming with the theoretical masses of 391.183 *m/z*, 462.220 *m/z* and 905.421 *m/z* (Fig. 2). All digestion products were in accordance to those of the model organism *E. coli*, suggesting *T. forsythia* to comprise a typical PGN composition of GlcNAc, MurNAc and attached stem peptides consisting of Ala, Glu and DAP.

Since the purity of the PGN preparations differed and the grade of digestion by mutanolysin was not determined, we do not claim quantification of different PGN building blocks. However, the observed major presence of G-M-tetra is in accordance with previous literature [19, 20].

### Determination of PGN stem peptide composition by MS^2^

**Fig. 3:**
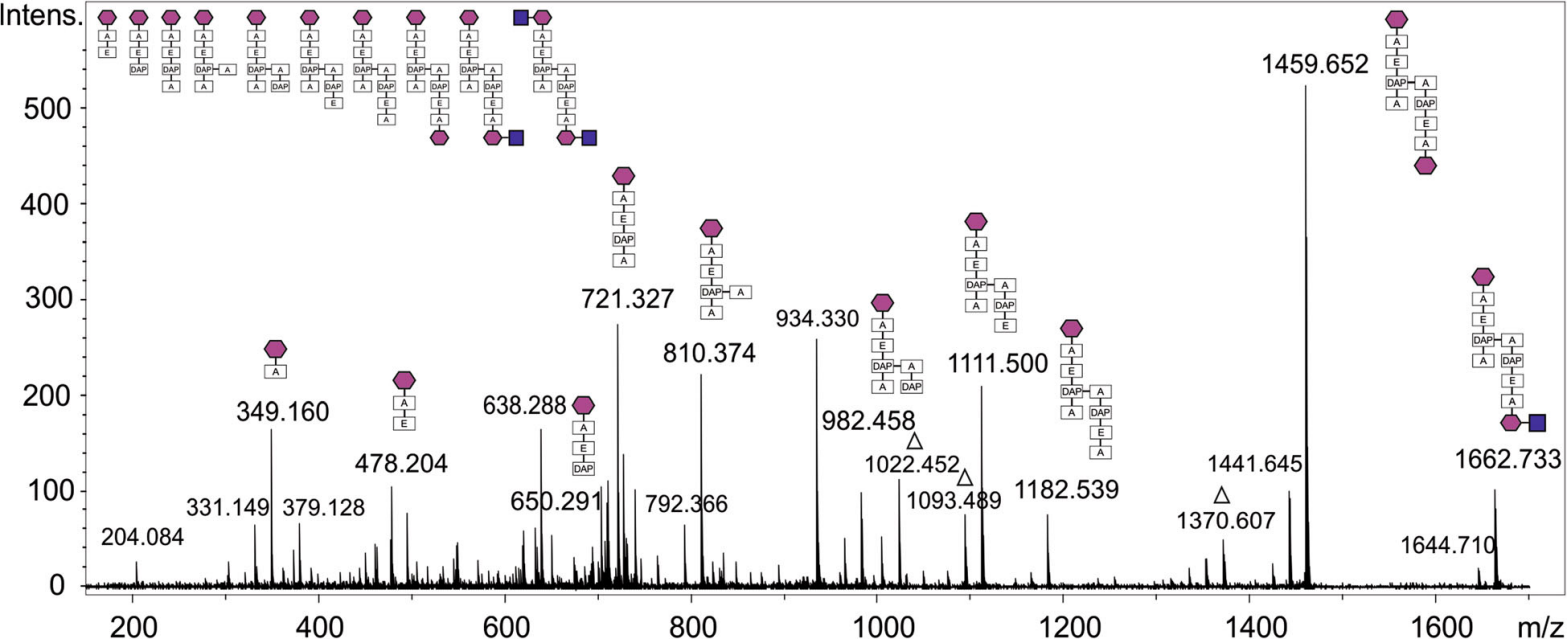
MS^*2*^ spectrum of mutanolysin digestion product GlcNAc-MurNAc-tetra-tetra-MurNAc-GlcNAc of *T. forsythia*, showing the arrangement of GlcNAc, MurNAc, Ala, Glu, DAP and Ala. Neutral losses of 18 Da and 89 Da (denoted by a triangle) were frequently observed. Legend: blue squares, GlcNAc; magenta hexagons, MurNAc, A, alanine; E, glutamic acid; DAP, diaminopimelic acid

To confirm the data obtained by MS analysis and elucidate the exact composition of the stem peptide and cross-linkage, the PGN building block G-M-tetra-tetra-M-G was analysed by MS^2^. Reduced mutanolysin digests were separated by RP-HPLC and the muropeptide-containing fraction was applied to LC-ESI-MS using a maXis 4G mass spectrometer (Bruker). MS^2^ analysis in positive ion mode was performed for the cross-linked G-M-tetra-tetra-M-G with a theoretical mass of 1865.813 *m/z*. The dimer was observed as a doubly charged ion with a mass of 933.410 *m/z* [M + 2H]^2+^ (Fig. 3). The fragmentation pattern showed the subsequent loss of GlcNAc, MurNAc, Ala, Glu, DAP, and Ala. This was a strong indication but not necessarily proof of the occurrence of a direct cross-linkage of DAP at the third position and Ala at the fourth position of opposing stem peptides. Notably, however, loss of an inner Ala was only observed subsequent to removal of DAP, which is supportive of the DAP-Ala cross-linkage.

According to these data, it is conceivable to assume for *T. forsythia* a typical PGN structure similar to that of *E. coli*, comprised of a glycan backbone and short peptides, arranged as Ala-Glu-DAP-Ala, forming a direct DAP-Ala cross-linkage, as typical of Gram-negative bacteria.

### Identification of the stereochemical variant of DAP

**Fig. 4:**
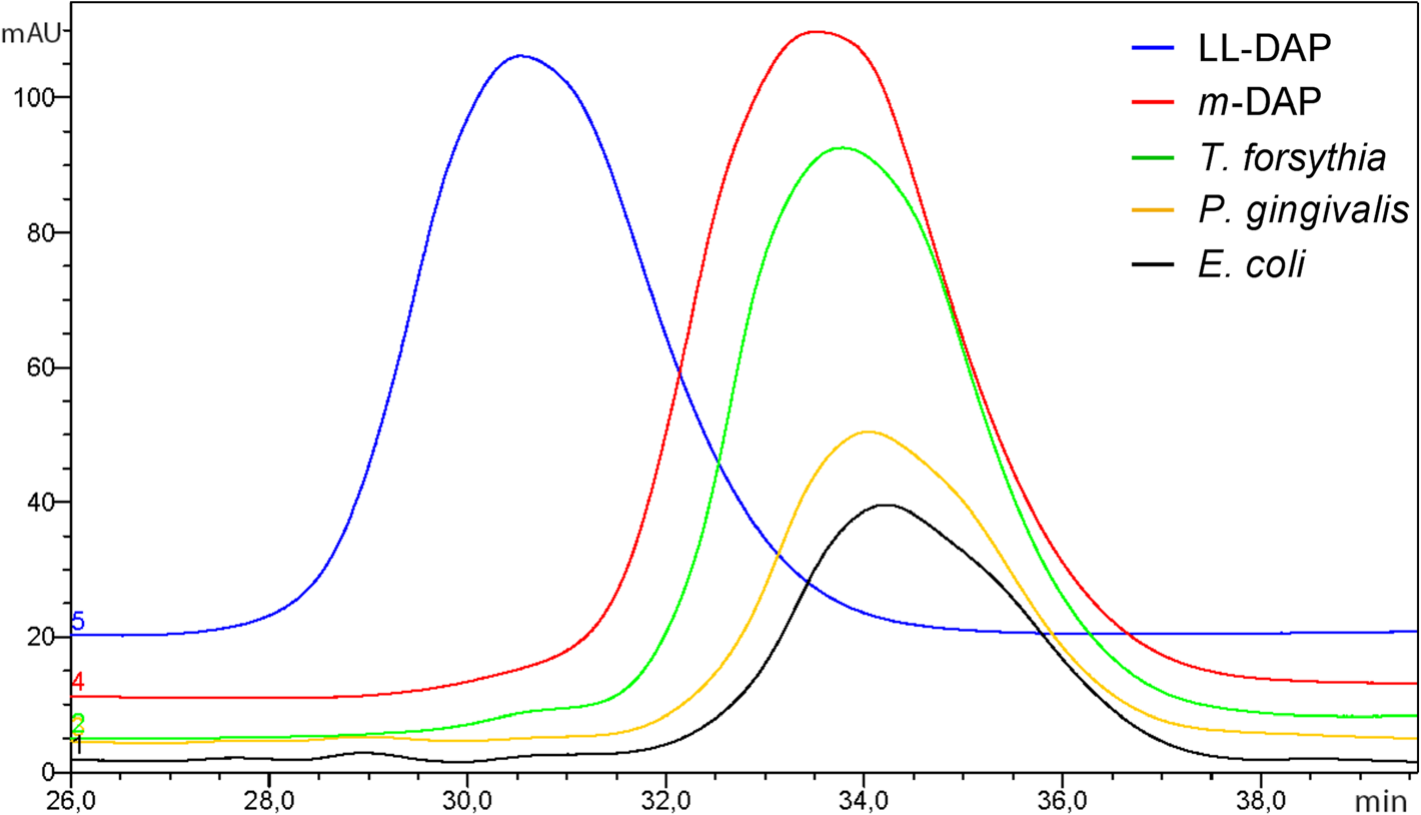
Separation of *m*-DAP and LL-DAP by reversed-phase HPLC after dabsylation, revealing the preponderance of *m*-DAP in all analysed peptidoglycan isolates. Overlay of chromatograms for *T. forsythia* peptidoglycan (green line), *P. gingivalis* peptidoglycan (yellow line) and *E. coli* peptidoglycan (black line) and the standards *m*-DAP (red line) and LL-DAP (blue line)

After PGN of *T. forsythia* had been proven to contain DAP as a component of the stem peptide, the question remained which stereochemical variant, *meso*- or LL-DAP, occurred. Standards were bought at Sigma, and PGN of *E. coli*, which contains *m*-DAP [30] and PGN of *P. gingivalis*, reported to contain LL-DAP [26], were used as references. PGN preparations were hydrolysed and dabsylated according to the method of Chang et al. [31] and applied to RP-HPLC, following the protocol for DAP isomer separation developed by Richaud et al. [32]. Dabsylated standards were separated, yielding peaks of approximately 90 mAU with a retention time of 33.5 min for *m*-DAP and 30.5 min for LL-DAP. Among strong signals of other amino acids and dabsylation reagent, a small, but unambiguous signal was obtained and revealed the predominance of *m*-DAP in all analysed samples, from *T. forsythia*, *E. coli,* and *P. gingivalis* (Fig. 4).

Considering all accomplished data, the PGN of *T. forsythia* has been determined as type A1γ. Additionally, the previously reported presence of LL-DAP in *P. gingivalis* must be revised and corrected to *m*-DAP.

## Discussion

Due to the lack of general enzymes of the de novo biosynthesis of PGN, the periodontal pathogen *T. forsythia* cannot synthesise PGN’s signature molecule MurNAc and depends on external MurNAc-sources for viability and maintenance of cell morphology [6–9]. However, using our newly developed, sensitive radioassay, the presence of MurNAc and anhMurNAc as a typical terminating residue of the sugar backbone strands of PGN in Gram-negative bacteria [16, 17] was unambiguously proven in *T. forsythia* cell walls. Mutanolysin digests of isolated PGN and subsequent LC-MS analysis revealed the typical PGN building blocks G-M-tripeptide, G-M-tetrapeptide, and the cross-linked G-M-tetra-tetra-M-G compound. Further digestion with the exo-*N*-acetylglucosaminidase NagZ and an amidase yielded single tri- and tetrapeptides (Fig. 2). All PGN digestion products were in accordance with those of *E. coli* PGN, revealing *T. forsythia* PGN to comprise the key components GlcNAc, MurNAc, Ala, Glu and DAP, as is typical of Gram-negative bacteria. These data were confirmed by an advanced MS^2^ analysis, which allowed the analysis of the cross-linked PGN building block GlcNAc-MurNAc-tetra-tetra-MurNAc-GlcNAc to contain the expected concatenation of GlcNAc, MurNAc, Ala, Glu, DAP, Ala, which indicates a direct cross-linkage between DAP on the third and Ala on the fourth position of opposite stem peptides, respectively. Gram-negative bacteria generally show a cross-linkage between stem peptides of two glycan strands via one amino group of the D-centre of *m*-DAP at position three and a carboxyl group of D-Ala at position 4. In *E. coli*, a second type of direct cross-linkage can occur between the L-centre of *m*-DAP of one strand and the D-centre of DAP of the other strand, as reported from tetra-tri or tri-tri compounds [33]. Regarding the PGN-type of *T. forsythia*, the remaining question concerned the stereochemical situation of the included DAP. This was clarified after dabsylation of standards and hydrolysed PGN material using RP-HPLC, revealing the preponderance of *m*-DAP. Considering all acquired data, PGN of *T. forsythia* could be determined as type A1γ [15]. At the current stage of analyses, it cannot be excluded that minor modifications, which are known to decorate some PGNs [34], might have escaped from detection.

Unexpectedly, the “red complex” bacterium *P. gingivalis*, initially planned to serve as a reference for LL-DAP-containing PGN, was shown to contain *m*-DAP. Barnard and Holt [26] reported previously that *P. gingivalis* contained LL-DAP and glycine, as determined by thin layer chromatography and amino acid analysis. The presence of LL-DAP together with an interpeptide bridge consisting of one or several glycine residues, responsible for an interpeptide bridge in the cross-linkage of PGN stem peptides, is characteristic of the PGN-type A3γ [15]. In the current study, we used a more sensitive HPLC-based method and clearly determined *m*-DAP, as well as typical PGN building blocks as revealed by MS analysis upon mutanolysin digest (Additional file 1: Figure S1) as known from PGN of *E. coli*. In most bacteria, *m*-DAP is incorporated into PGN, however, in some bacteria, mostly Gram-positives, LL-DAP is found [15]. The presence of *m*-DAP instead of LL-DAP in *P. gingivalis* PGN is supported by the observation that *m*-DAP is generated via a unique dehydrogenase pathway in both, *T. forsythia* and *P. gingivalis*; the putative *T. forsythia m*-DAP-dehydrogenase Tanf_04065 and the biochemically proven *P. gingivalis m*-DAP-dehydrogenase PG_0806 [35] show 59% identity within their amino acid sequence. In this pathway 2-amino-6-oxopimelate, generated from 2,3,4,5-tetrahydrodipicolinate, is directly converted in the dehydrogenase-catalysed reaction to *m*-DAP. This pathway does not proceed via LL-DAP. In *E. coli* and most other bacteria, DAP is produced in the course of lysine biosynthesis, whereby initially LL-DAP is generated by the succinyl-diaminopimelate desuccinylase DapE [36] and subsequently converted into *m*-DAP by the diaminopimelate epimerase DapF [37, 38], which is a direct precursor of L-lysine. According to the KEGG database, *P. gingivalis* lacks the DapE and DapF enzymes, pinpointing a direct production of *m*-DAP via the recently characterized diaminopimelate dehydrogenase PG_0806 [35]. Stone et al. reported on PG_0806 in the context of new treatments against periodontal disease, since knock-out of the corresponding gene was lethal to cells and the direct *m*-DAP producing pathway is limited to oral pathogens such as *P. gingivalis* and *T. forsythia*, excluding health-associated oral bacteria. According to these data altogether, the classification of *P. gingivalis* PGN needs to be revised to type A1γ. However, it should be noted that in *T. forsythia*, the situation concerning the genetic make-up for DAP biosynthesis is less straightforward, which supports the importance of the present study. Besides the presence of the diaminopimelate dehydrogenase Tanf_04065 leading to *m*-DAP as described above, the bacterium has a predicted DAP-aminotransferase DapL (Tanf_03505 in *T. forsythia*), which could convert 2,3,4,5-tetrahydrodipicolinate directly into LL-DAP. Redundant pathways for the biosynthesis of DAP have been reported, for instance, in *Bacteroides fragilis* – also a member of the *Bacteroidetes* family of bacteria as *T. forsythia* – and in *Clostridium thermocellum* [39]. Importantly, both of these bacteria have a DapF enzyme for the subsequent conversion of LL-DAP into *m*-DAP as immediate precursor of L-lysine. Such an enzyme is missing in the *T. forsythia* genome, making the aminotransferase LL-DAP pathway ineffective for lysine biosynthesis. Of note, DapF would have been easily recognizable in silico; DapF epimerases belong to the protein family PF01678 (pfam identifier; EMB_EBI database) that only contains known or predicted Dap epimerases.

Generally, the DAP dehydrogenase pathway is regarded as an ancillary mechanism for DAP biosynthesis and for *Corynebacterium glutamicum* it was hypothesised that especially the ammonium concentration in the culture medium affects a switch between DAP biosynthesis pathways [40, 41]. If this is also valid for *T. forsythia* and if a switch between DAP pathways under different conditions would be manifested in the PGN composition needs to be investigated.

## Conclusion

In the course of this study, the basic structure of the bacterial key molecule peptidoglycan was identified for the periodontal pathogen *T. forsythia*. Despite the presence of both a DAP-dehydrogenase and a DAP-aminotransferase orthologue in the bacterial genome, only *m*-DAP synthesised via the DAP-dehydrogenase pathway was detected in the PGN. The unusual PGN metabolism of *T. forsythia,* employing alternate routes for the synthesis of essential PGN precursors, opens new perspectives on the bacterial cell wall metabolism, where PGN constitutes a main target for antibacterial drugs, since destruction or manipulation of its biosynthesis disturbs cell viability. Knowledge about structural properties of PGN of two “red complex” bacteria, may assist progress of novel strategies for the development of antibacterial treatment of periodontal disease.

## Methods

### Bacterial strains and growth conditions

*Tannerella forsythia* ATCC 43037 and *Porphyromonas gingivalis* W83 were obtained from the American Type Culture Collection (Manassas, VA, United States). Cultivation was done anaerobically at 37 °C in brain heart infusion medium (37 g l^− 1^; Oxoid, Basingstoke, United Kingdom), supplemented with yeast extract (10 g l^− 1^; Sigma, Vienna, Austria), L-cysteine (1 g l^− 1^; Sigma), hemin (5 mg ml^− 1^; Sigma) and menadione (2 mg ml^− 1^; Sigma). For *T. forsythia*, 5% (v/v) horse serum (Thermo Fisher Scientific, Vienna, Austria) and MurNAc (20 μg ml^− 1^; Carbosynth, Compton, United Kingdom) were added.

*Escherichia coli* DH5α was cultivated in Luria Bertani broth (Miller’s LB broth base; Thermo Fisher Scientific) at 37 °C with shaking.

### Isolation of peptidoglycan

PGN was isolated essentially following a published procedure [29]. Briefly, biomass was harvested from 1 l of culture grown to the stationary phase by centrifugation (5000 g, 30 min, 4 °C), resuspended in 60 ml distilled water and transferred drop-wise into 65 ml of boiling 8% sodium dodecyl sulfate (SDS; Sigma) under constant stirring to lyse cells. The suspension was further boiled for 1 h, reduced to the former volume using a rotary evaporator, and stirred overnight. SDS was removed by several washing steps with distilled water, 60 ml, each, using an Optima L-100XP ultracentrifuge from Beckman Coulter (rotor Ti70, 35,000 rpm, 30 min, 40 °C) followed by dialysis against distilled water for 4 days at room temperature. For the total volume of 12 ml of that PGN solution, 200 μl of an α-amylase solution (24 mg ml^− 1^; Sigma) were added and the mixture was incubated at 37 °C for 2 h under constant shaking. Further, 320 μl of pre-incubated Pronase E solution (10 mg ml^− 1^ in 10 mM Tris-HCl, pH 7.5; Sigma) was added and incubated at 60 °C for 1.5 h. Preparations were washed, boiled for 1 h, washed again, and dried in a Speed Vac vacuum centrifuge (Thermo Fisher Scientific, Vienna, Austria).

### Identification of MurNAc and anhMurNAc in *T. forsythia* cell walls by radioactive labelling

Lyophilized *T. forsythia* cells were resuspended in 40 μl of 2-(N-morpholino) ethanesulfonic acid (MES) buffer, pH 6.0, and digested with 10 μg of the *N*-acetylmuramidase mutanolysin from *Streptomyces globisporus* (Sigma) overnight at 37 °C. Afterwards, *Bacillus subtilis N*-acetylglucosaminidase NagZ [42] and *E. coli* amidase AmiD [43], 10 μg, each, were added and the mixture was incubated for further 2 h at 37 °C. The digested sample was centrifuged and the supernatants were incubated in a reaction mixture containing 50 mM Tris-HCl, pH 7.6, 10 mM MgCl_2_ and γ-^32^P-ATP (140 kBq; Hartmann Analytic, Braunschweig, Germany; specific activity: 111 TBq (3000 Ci)/ mmol). The labelling reactions were started by the addition of the specific MurNAc/GlcNAc kinase MurK of *Clostridium acetobutylicum* [27] or *E. coli* anhMurNAc kinase AnmK [28], respectively, 20 ng, each, and spotted immediately (time point 0) and after 1 h of incubation at 37 °C on a Silica 60 F_254_ thin-layer chromatography (TLC) plate (20 cm × 20 cm; Merck, Darmstadt, Germany). Reaction products were separated in a basic solvent of n-butyl alcohol/methanol/25% ammonium hydroxide (w/v)/water (5:4:2:1) and radioactive products were detected using a Typhoon TRIO + biomolecular imager (GE Healthcare).

### Analysis of peptidoglycan fragments by LC-MS

PGN (0.5 mg) was resuspended in 70 μl of sodium phosphate buffer (200 mM, pH 6.0) and digested with mutanolysin from *Streptomyces globisporus* (50 μg ml^− 1^; Sigma). After incubation over night at 37 °C under constant shaking, the reaction was stopped by heating at 100 °C for 25 min. To produce single peptides, PGN was further digested with 50 μg ml^− 1^ of *N*-acetylglucosaminidase NagZ [42] for 6 h and 100 μg ml^− 1^ of amidase AmiE [42] overnight. Muropeptides were reduced by mixing 100 μl of the digest with 100 μl sodium borate buffer (0.5 M, pH 9.0) and adding 5 mg of sodium borohydride. After incubation for 30 min at room temperature, the reaction was stopped with 5–10 μl 20% phosphoric acid, adjusting the pH to 3.5. After centrifugation (12,000 g, 10 min, room temperature), the supernatant was dried in a Speed Vac vacuum centrifuge (Thermo Fisher Scientific) and dissolved in 50 μl of distilled water. Preparation aliquots of 5 μl were applied to HPLC at a flow rate of 0.2 ml min^− 1^ and an elution profile (using buffer A: formic acid with 0.05% ammonium formate, and buffer B: 100% acetonitrile) as described previously [44]. LC-ESI-MS measurements were performed using a Gemini C18 column (150 × 4.6 mm, 110 Å, 5 μm; Phenomenex) and an UltiMate 3000 RS HPLC system (Dionex) coupled to a MicrO-TOF II mass spectrometer (Bruker), operated in positive ion mode.

### Analysis of peptidoglycan fragments by MS^2^

PGN (0.5 mg) was digested with mutanolysin (as described above) followed by reduction using sodium borohydride at a final concentration of 8 μg ml^− 1^ in sodium borate buffer (400 mM, pH 10.0). The reaction was stopped after 15 min by acidifying the solution with 1 to 2 drops of 4 M acetic acid. Purification and desalting of the sample was performed via a reversed-phase SPE column (Strata C18-E, 50 mg; Phenomenex), equilibrated with 80 mM formic acid, buffered to pH 3.0 with ammonia (solvent A), and elution was done with 80% with acetonitrile in sodium borate buffer. Samples were dried in a Speed Vac vacuum centifuge, resolved in 200 μl of solvent A, and analysed by HPLC (Nexera X2, Shimadzu, Korneuburg, Austria). Separation was performed on a Hyperclone ODS column (250 cm × 4 mm, 5 μm particle size; Phenomenex) at a flow rate of 1 ml min^− 1^, at 35 °C, with a fraction size of 0.5 ml and UV detection at 215 nm. After an initial 10 min holding of 1% solvent B (80% acetonitrile in solvent A), a linear gradient from 1 to 25% B over 30 min was applied. The muropeptide-containing HPLC fraction (based on the UV signal in combination with MS screening) was subjected to LC-ESI-MS analysis using a BioBasic C18 column (320 μm × 150 mm, 5 μm; Thermo Fisher Scientific), an UltiMate 3000 Nano LC system (Dionex) and a maXis 4G mass spectrometer (Bruker). A linear gradient from 1 to 50% solvent B over 11 min ascending to 85% B in 4 min was applied at a flow rate of 600 nl min^− 1^. After acquiring LC-MS data in a full scan, multiple reaction monitoring experiment of 933.4 *m/z* was performed with different collision energies (35, 45 and 50 eV) in positive ion mode. Data interpretation was done with DataAnalysis 4.0 (Bruker, Bremen, Germany).

### Determination of the stereochemistry of DAP

Separation of DAP isomers was performed according to Richaud et al. [32]. Isolated PGN of *T. forsythia*, *E. coli*, and *P. gingivalis* were hydrolysed with 6 N HCl containing 0.2% thioglycolic acid at 110 °C for 16 h. Samples were dried using a nitrogen evaporator and washed with distilled water for three times. *m*-DAP and LL-DAP were purchased from Sigma and used as standards. Dabsylation was performed by the method of Chang et al. [31], using 100 μg of samples or standards dissolved in 100 μl 0.1 M sodium bicarbonate buffer, pH 9.0. 200 μl of dabsyl chloride (4 nmol μl^− 1^; Sigma) were added and samples were incubated at 70 °C for 20 min. Dried preparations were dissolved in 100–500 μl ethanol (70%, v/v) and 20 μl were injected onto a reversed-phase HPLC column (Ultimate 3000, C18, 150 × 4.6 mm). An isocratic elution was done at 37 °C with 12 mM ammonium phosphate, pH 6.5-acetonitrile-dimethylformamide (69:27:4, vol/vol/vol) at a flow rate of 0.6 ml min^− 1^ and detection was done at 436 nm.

## Additional file

Additional file 1: **Figure S1.** LC-MS analysis of *P. gingivalis* PGN digests with **(A)** mutanolysin yielding G-M-peptides and cross-linked G-M-tetra-tetra-M-G and **(B)** NagZ and amidase, yielding single peptides. Legend: TIC, total ion chromatogram; G, GlcNAc; M, MurNAc. (DOCX 335 kb)

## Data Availability

The datasets used and/or analysed during the current study are available from the corresponding author on reasonable request.

## References

[1] Tanner AC, Izard J (2006). *Tannerella forsythia*, a periodontal pathogen entering the genomic era. Periodontol 2000.

[2] Tanner ACR, Listgarten MA, Ebersole JL, Strezempko MN (1986). *Bacteroides forsythus* sp. nov., a slow-growing, fusiform *Bacteroides* sp. from the human oral cavity. Int J Syst Bacteriol.

[3] Slots J, Listgarten MA (1988). *Bacteroides gingivalis, Bacteroides intermedius* and *Actinobacillus actinomycetemcomitans* in human periodontal diseases. J Clin Periodontol.

[4] Holt SC, Ebersole JL (2005). *Porphyromonas gingivalis*, *Treponema denticola*, and *Tannerella forsythia*: the “red complex”, a prototype polybacterial pathogenic consortium in periodontitis. Periodontol 2000.

[5] Hajishengallis G, Lamont RJ (2012). Beyond the red complex and into more complexity: the polymicrobial synergy and dysbiosis (PSD) model of periodontal disease etiology. Mol Oral Microbiol.

[6] Wyss C (1989). Dependence of proliferation of *Bacteroides forsythus* on exogenous *N*-acetylmuramic acid. Infect Immun.

[7] Hottmann I, Mayer VMT, Tomek MB, Friedrich V, Calvert MB, Titz A (2018). *N*-Acetylmuramic acid (MurNAc) auxotrophy of the oral pathogen *Tannerella forsythia*: characterization of a MurNAc kinase and analysis of its role in cell wall metabolism. Front Microbiol.

[8] Friedrich V, Pabinger S, Chen T, Messner P, Dewhirst FE, Schäffer C (2015). Draft genome sequence of *Tannerella forsythia* type strain ATCC 43037. Genome Announc.

[9] Gisin J, Schneider A, Nägele B, Borisova M, Mayer C (2013). A cell wall recycling shortcut that bypasses peptidoglycan *de novo* biosynthesis. Nat Chem Biol.

[10] Ruscitto A, Sharma A (2018). Peptidoglycan synthesis in *Tannerella forsythia*: scavenging is the modus operandi. Mol Oral Microbiol.

[11] Ruscitto A, Hottmann I, Stafford GP, Schäffer C, Mayer C, Sharma A (2016). Identification of a novel *N*-acetylmuramic acid transporter in *Tannerella forsythia*. J Bacteriol.

[12] Ruscitto A, Honma K, Veeramachineni VM, Nishikawa K, Stafford GP, Sharma A (2017). Regulation and molecular basis of environmental muropeptide uptake and utilization in fastidious oral anaerobe *Tannerella forsythia*. Front Microbiol.

[13] Vollmer W, Seligman SJ (2010). Architecture of peptidoglycan: more data and more models. Trends Microbiol.

[14] Vollmer W, Bertsche U (2008). Murein (peptidoglycan) structure, architecture and biosynthesis in *Escherichia coli*. Biochim Biophys Acta.

[15] Schleifer KH, Kandler O (1972). Peptidoglycan types of bacterial cell walls and their taxonomic implications. Bacteriol Rev.

[16] Höltje JV, Mirelman D, Sharon N, Schwarz U (1975). Novel type of murein transglycosylase in *Escherichia coli*. J Bacteriol.

[17] Harz H, Burgdorf K, Höltje JV (1990). Isolation and separation of the glycan strands from murein of *Escherichia coli* by reversed-phase high-performance liquid chromatography. Anal Biochem.

[18] Vollmer W, Blanot D, de Pedro MA (2008). Peptidoglycan structure and architecture. FEMS Microbiol Rev.

[19] Vollmer W, Höltje JV (2004). The architecture of the murein (peptidoglycan) in gram-negative bacteria: vertical scaffold or horizontal layer(s)?. J Bacteriol.

[20] Glauner B (1988). Separation and quantification of muropeptides with high-performance liquid chromatography. Anal Biochem.

[21] Barnickel G, Labischinski H, Bradaczek H, Giesbrecht P (1979). Conformational energy calculation on the peptide part of murein. Eur J Biochem.

[22] Dmitriev BA, Ehlers S, Rietschel ET (1999). Layered murein revisited: a fundamentally new concept of bacterial cell wall structure, biogenesis and function. Med Microbiol Immunol.

[23] Meroueh SO, Bencze KZ, Hesek D, Lee M, Fisher JF, Stemmler TL (2006). Three-dimensional structure of the bacterial cell wall peptidoglycan. Proc Natl Acad Sci U S A.

[24] Tinelli R, Pillot J (1966). Etude de la composition du glycopeptide de *Treponema reiteri*. Compt Rend Acad Sci (Paris).

[25] Kato K, Umenotoh T, Sagawa H, Kotani S (1979). Lanthionine as an essential constituent of cell wall peptidoglycan of Fusobacterium nucleatum. Curr Microbiol.

[26] Barnard MR, Holt SC (1985). Isolation and characterization of the peptidoglycans from selected gram-positive and gram-negative periodontal pathogens. Can J Microbiol.

[27] Reith J, Berking A, Mayer C (2011). Characterization of an *N*-acetylmuramic acid/*N*-acetylglucosamine kinase of *Clostridium acetobutylicum*. J Bacteriol.

[28] Uehara T, Suefuji K, Valbuena N, Meehan B, Donegan M, Park JT (2005). Recycling of the anhydro*-N-*acetylmuramic acid derived from cell wall murein involves a two-step conversion to *N*-acetylglucosamine-phosphate. J Bacteriol.

[29] Desmarais SM, Cava F, de Pedro MA, Huang KC (2014). Isolation and preparation of bacterial cell walls for compositional analysis by ultra performance liquid chromatography. J Vis Exp.

[30] Van Heijenoort J, Elbaz L, Dezelee P, Petit JF, Bricas E, Ghuysen JM (1969). Structure of the meso-diaminopimelic acid containing peptidoglycans in *Escherichia coli* B and *Bacillus megaterium* KM. Biochemistry..

[31] Chang JY, Knecht R, Braun DG (1981). Amino acid analysis at the picomole level. Application to the C-terminal sequence analysis of polypeptides. Biochem J.

[32] Richaud C, Higgins W, Mengin-Lecreulx D, Stragier P (1987). Molecular cloning, characterization, and chromosomal localization of dapF, the *Escherichia coli* gene for diaminopimelate epimerase. J Bacteriol.

[33] Glauner B, Höltje JV, Schwarz U (1988). The composition of the murein of *Escherichia coli*. J Biol Chem.

[34] Espaillat A, Forsmo O, El Biari K, Bjork R, Lemaitre B, Trygg J (2016). Chemometric analysis of bacterial peptidoglycan reveals atypical modifications that empower the cell wall against predatory enzymes and fly innate immunity. J Am Chem Soc.

[35] Stone VN, Parikh HI, El-rami F, Ge X, Chen W, Zhang Y (2015). Identification of small-molecule inhibitors against meso-2, 6-diaminopimelate dehydrogenase from *Porphyromonas gingivalis*. PLoS One.

[36] Kindler SH, Gilvarg C (1960). *N*-Succinyl-L-2,6-diaminopimelic acid deacylase. J Biol Chem.

[37] Wiseman JS, Nichols JS (1984). Purification and properties of diaminopimelic acid epimerase from *Escherichia coli*. J Biol Chem.

[38] Mengin-Lecreulx D, Michaud C, Richaud C, Blanot D, van Heijenoort J (1988). Incorporation of LL-diaminopimelic acid into peptidoglycan of *Escherichia coli* mutants lacking diaminopimelate epimerase encoded by *dapF*. J Bacteriol.

[39] Hudson AO, Klartag A, Gilvarg C, Dobson RC, Marques FG, Leustek T (2011). Dual diaminopimelate biosynthesis pathways in *Bacteroides fragilis* and *Clostridium thermocellum*. Biochim Biophys Acta.

[40] Wehrmann A, Phillipp B, Sahm H, Eggeling L (1998). Different modes of diaminopimelate synthesis and their role in cell wall integrity: a study with *Corynebacterium glutamicum*. J Bacteriol.

[41] Hartmann M, Tauch A, Eggeling L, Bathe B, Möckel B, Pühler A (2003). Identification and characterization of the last two unknown genes, *dapC* and *dapF*, in the succinylase branch of the L-lysine biosynthesis of *Corynebacterium glutamicum*. J Biotechnol.

[42] Litzinger S, Duckworth A, Nitzsche K, Risinger C, Wittmann V, Mayer C (2010). Muropeptide rescue in *Bacillus subtilis* involves sequential hydrolysis by β-*N*-acetylglucosaminidase and *N*-acetylmuramyl-L-alanine amidase. J Bacteriol.

[43] Uehara A, Takada H (2007). Functional TLRs and NODs in human gingival fibroblasts. J Dent Res.

[44] Borisova M, Gisin J, Mayer C (2014). Blocking peptidoglycan recycling in *Pseudomonas aeruginosa* attenuates intrinsic resistance to fosfomycin. Microb Drug Resist.

