# Supplementary material for: Peptidoglycan-type analysis of the N-acetylmuramic acid auxotrophic oral pathogen Tannerella forsythia and reclassification of the peptidoglycan-type of Porphyromonas gingivalis
Source: BMC Microbiol. 2019 Sep 2;19:200. doi: 10.1186/s12866-019-1575-7 (PMC6721243; doi:10.1186/s12866-019-1575-7)
Supplement: Supplementary file 1 — Figure S1. LC-MS analysis of P. gingivalis PGN digests with (A) mutanolysin yielding G-M-peptides and cross-linked G-M-tetra-tetra-M-G and (B) NagZ and amidase, yielding single peptides. Legend: TIC, total ion chromatogram; G, GlcNAc; M, MurNAc. (DOCX 335 kb) [file 12866_2019_1575_MOESM1_ESM.docx]

**Supplemental figure of Valentina M. T. Mayer *et al.***


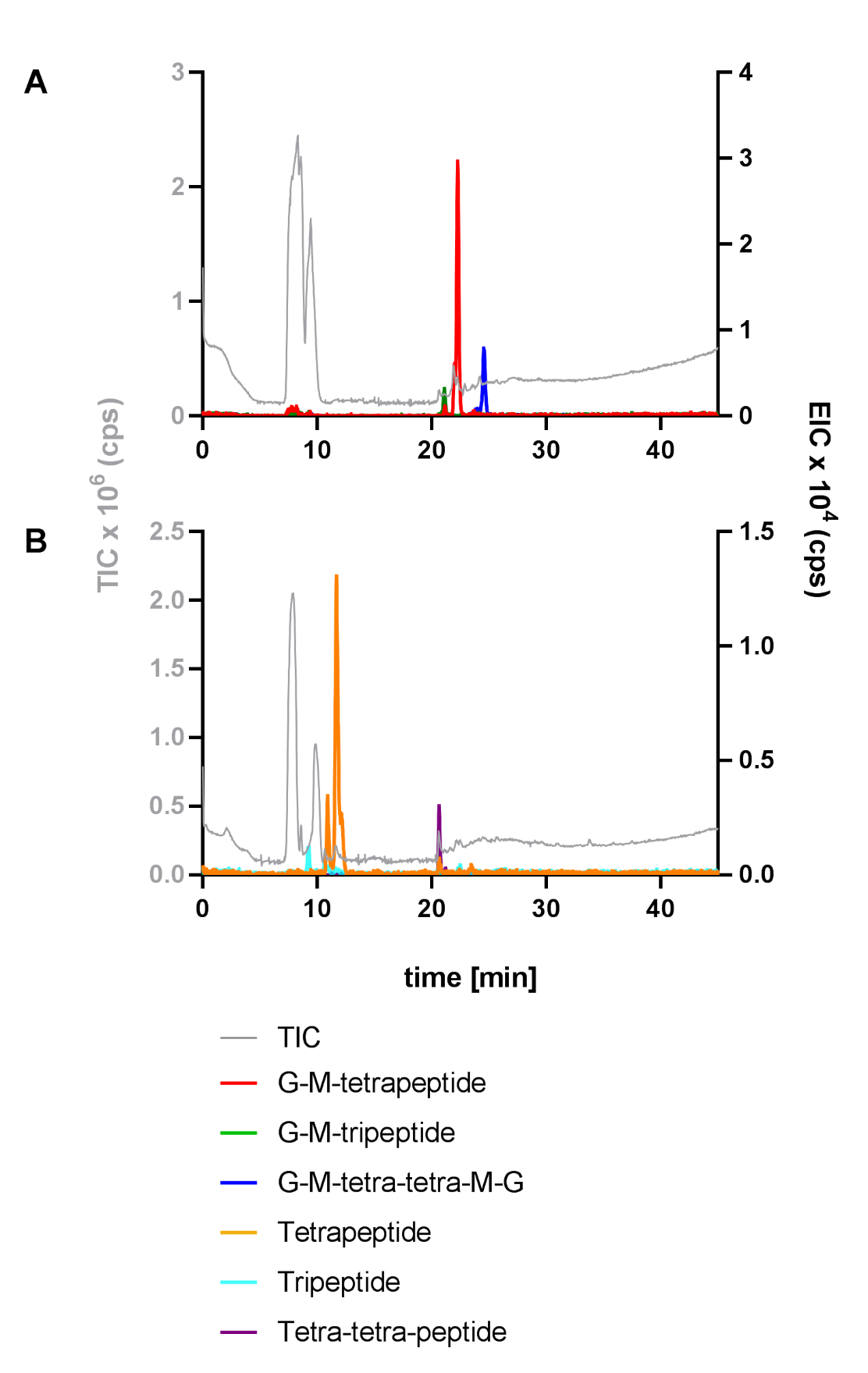


**Fig. S1** LC-MS analysis of P. gingivalis PGN digests with **(A)** mutanolysin yielding G-M-peptides and cross-linked G-M-tetra-tetra-M-G and **(B)** NagZ and amidase, yielding single peptides. Legend: TIC, total ion chromatogram; G, GlcNAc; M, MurNAc.
